# Demographic Factors and Cognitive Function Assessments Associated with Mild Cognitive Impairment Progression for the Elderly

**DOI:** 10.1155/2020/3054373

**Published:** 2020-02-08

**Authors:** Hong-yun Qin, Xu-dong Zhao, Bing-gen Zhu, Cheng-ping Hu

**Affiliations:** Department of Psychiatry, Shanghai Pudong New Area Mental Heath Center, Tongji University School of Medicine, Shanghai 200124, China

## Abstract

**Objectives:**

In this study, we aimed to conduct a 6-year follow-up and acquire a large sample dataset to analyze the most important demographic factors and cognitive function scale variables associated with mild cognitive impairment (MCI) progression for an elderly cohort (age ≥ 60 years old). *Patients and Methods.* We analyzed the subjects who had participated in a survey in 2011 and were successfully contacted in the later survey in 2017. For each subject, the basic demographic information was recorded, including sex, age, education level, marital status, working status, income level, and physical mental illness history. Cognitive assessments were performed using the following scales if possible: (1) the mini-mental state examination (MMSE) scale, (2) Montreal cognitive assessment (MoCA), (3) the clinical dementia rating (CDR) scale, and (4) Hamilton Depression Scale (HAMD-17).

**Results:**

The progression outcomes were different between sexes, among age brackets, education degrees, occupations types, and income levels; different progression groups had distinct children numbers (*p* < 0.001), heights (*p* < 0.001), heights (*p* < 0.001), heights (*p* < 0.001), heights (

**Conclusions:**

In conclusion, the MCI progression outcomes were associated with sex, age, education degrees, occupations types, income level, children number, height, and weight. MoCA and MMSE scales are supporting tools to predict the progression outcomes, especially combined with the demographic data.

## 1. Introduction

For aged population, cognitive impairment has a high prevalence and affects people worldwide. Throughout the past decades, studies have shown various risk factors that play roles for the development of cognitive impairment, such as hypercholesterolemia, smoking, other cardiovascular risk factors, and anxiety/depression [1–4]. Recent studies found that cognitive impairment in the elderly is strongly affected by cognitive function in middle age or the early period of old age [5]. The lower cognitive function score in middle age implied a higher risk of mild cognitive impairment (MCI) or dementia in later life. MCI involves cognitive decline (including memory loss or non-memory-related cognitive symptoms) that is greater than that which occurs in normal aging, with some limitations to daily function [6]. Generally, these patients with objective memory impairment do not meet the accepted criteria for dementia [7]. MCI is a precursor to multiple types of dementia. For example, 10–15% of patients with MCI may develop dementia each year [8]. It is important to prevent the MCI-dementia development, in due course. Theoretically, demographic data and cognition assessment scales may provide early signs for MCI-progression prediction. Our pilot analysis and some published work suggested that the progression of MCI to dementia is not only related to aging and vascular risk factors but also related to low total scores of several cognition tests years ago [9–12]. In this study, we aimed to conduct a 6-year follow-up and acquire a large sample dataset to analyze the most important demographic factors and cognitive function scale variables associated with MCI progression for the elderly.

## 2. Patients and Methods

### 2.1. Subjects

This study was approved by the ethical committee of Tongji University and carried out in Pudong New area in Shanghai. From June 2011 to June 2012 (no. PKJ2010-Y26), we had recorded information of an elderly cohort (age ≥ 60 years old), and only those successfully contacted in the later survey (in 2017) were enrolled for analysis. All participants have signed informed consent. Only those with enough audiovisual level to complete the necessary examinations were included. The basic demographic information of each subject was recorded, including sex, age, education level, marital status, working status, income level, and other mental illness history.

### 2.2. MCI and Dementia Diagnostic Criteria

MCI was diagnosed using the revised Mayo Clinic criteria [13, 14]: (1) the elderly consciously exhibited memory loss, especially those with memory impairment for more than 3 months; (2) the overall cognitive function is normal through the mini-mental state examination (MMSE total score: illiterate subjects > 17, with primary school education > 20 points, and others > 24 point); (3) the clinical dementia rating (CDR) score reached a level of 0.5; (4) Montreal cognitive assessment scale: MoCA score ≤ 26; (5) with normal function of daily life; (6) the patient did not meet the diagnostic criteria for dementia.

Here, the criteria for dementia were as follows [15, 16]: MMSE test: illiterate subjects ≤17, subjects with primary school education ≤20 points, and subjects with education of middle school or above ≤24 points. Those with definite blindness or speech difficulties were excluded.

### 2.3. Cognitive Assessments

Subjects received the cognitive assessments using the following scales: (1) the mini-mental state examination (MMSE) scale [17], (2) Montreal cognitive assessment (MoCA) Chinese version, (3) the clinical dementia rating (CDR) scale [18], and (4) Hamilton Depression Scale (HAMD-17) [19, 20].

### 2.4. Statistical Analysis

Statistical analysis was performed by SPSS 19.0 software (IBM, New York). Quantitative data were first tested the normality using the one-sample Kolmogorov–Smirnov test (1-sample KS test) and expressed as mean ± standard deviation (SD) or median with the interquartile range if not normally distributed. Quantitative data were compared between the two groups using the *t*-test (in normal distribution) or a nonparametric test (Mann–Whitney test, not in normal distribution) and one-way ANOVA was used for comparison of three groups. The chi-squared test was performed to compare the frequencies of categorical data. The receiver operating characteristic (ROC) curves of the interested variables were drawn using SPSS. *p* < 0.05 was considered statistically significant.

## 3. Results

### 3.1. Clinical Features of Enrolled Subjects

As shown in Table 1, we have originally recorded 2901 subjects in 2011. Six years later, 1648 participants were out of contact in follow-up, and 1253 cases were traced but 32 failed to complete the required scales or provide necessary general information. Finally, 1229 cases were successfully recorded in 2017. Among this cohort, there were 58 dementia cases, 441 MCI ones, and 730 healthy ones, while in 2017, there were 170 dementia ones, 975 MCI ones, and only 84 healthy ones. These data suggest that dementia and MCI develop rapidly in the elderly. Additionally, we analyzed differences in the features of the lost cohort and the followed-up cohort. The lost group had significantly higher age (*p* < 0.01), larger single proportion (*p* < 0.01), fewer children (*p* < 0.01), and higher income levels (*p* < 0.01). For other physical indexes, there were no significant differences in height, weight, memory loss complaints, drinking history, smoking history, personality propensity, hyperlipidemia, hypertension, diabetes, family history of dementia, and so on.

### 3.2. Progression Overview of MCI and Dementia

**Figure 1:**
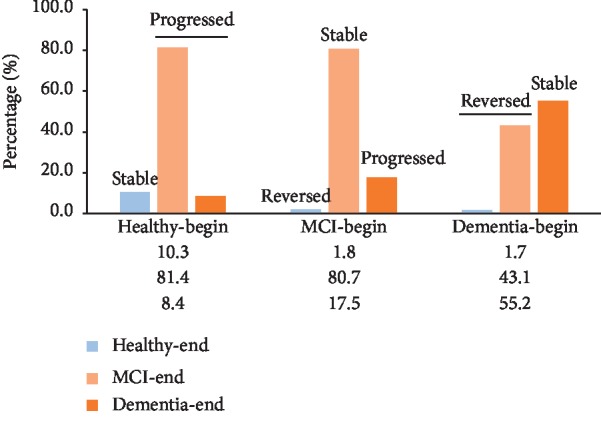
Progression overview of MCI and dementia. Six years later, the progression was evaluated, and the percentages of three subtypes in the end were expressed for each classification diagnosed in 2011. The 730 healthy ones were diagnosed: 75 healthy ones, 594 MCI, and 61 dementia patients; the 441 MCI participants developed into: 8 healthy ones, 356 MCI and 77 patients; the 58 dementias developed into: 1 healthy subject, 25 MIC patients and 32 dementias.

Six years later, the progression was evaluated. There were 58 dementia, 441 MCI, and 730 healthy cases in 2011, and they were largely diversified (Figure 1). The 730 healthy ones were diagnosed as follows in 2017: 75 healthy ones, 594 MCI, and 61 dementia patients; the 441 MCI participants developed into 8 healthy ones, 356 MCI, and 77 patients; the 58 dementia ones developed into: 1 healthy subject, 25 MIC patients, and 32 dementia. For both the healthy and MCI cohorts, they result in more than 80% MCI population, and only 10% of the healthy cohort escaped from cognition impairment (Figure 1), which suggests that there is a strong trend toward MCI (or even dementia) development for the elderly population.

### 3.3. Risk Factors regarding Cognition Impairment Progression

**Figure 2:**
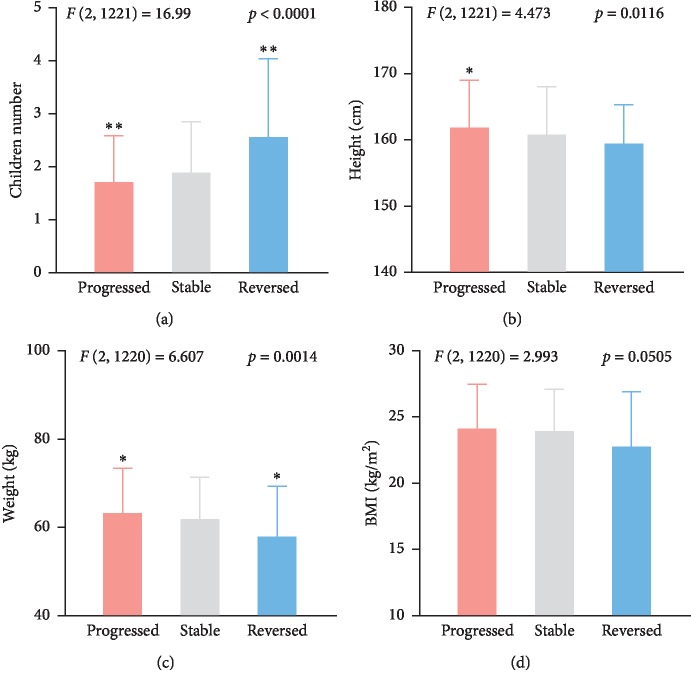
Progression outcomes were associated with children number, height and height. (a) Patients with positive outcomes had more children. (b) Patients with positive outcomes had lower heights. (c) Patients with positive outcomes had lower weight. (d) BMI levels were slightly correlated with progression outcomes. ^*∗*^*p* < 0.05 vs Stable.

Next, we divided all the cases into three groups according to the development direction (Figure 1): reversed, stable, and progressed. Progression referred to a change of healthy into MCI or MCI into dementia. The demographic, habit, and scale factors were analyzed to reveal the most important roles during cognition impairment progression. First, there were significant differences in progression outcomes between sexes (*χ*^2^ = 14.1, *p*=0.001) (Table 2); that is, males had higher risk in cognition impairment progression in comparison with females. Afterwards, the trends of cognition impairment showed distinct features among age brackets. All subjects were divided into five age brackets (brackets 1–5: 50–59, 60–69, 70–79, 80–89, 90–99). The cohorts with lower ages were more likely to have progression outcomes; that is, brackets 1-2 had significantly higher risks of progression (*χ*^2^ = 29.247, *p* < 0.0001) (Table 3), which suggests that the early period of the elderly stage is a sensitive period of cognition impairment onset. In detail, the proportion of progression sharply dropped during 66 and 67 years (66.2% for 66 years vs 57.8% for 67 years). Accordingly, it is reasonable to take precaution of cognition impairment in the susceptible period (below 66 years). Furthermore, the progression outcomes were correlated with the education degree (*χ*2 = 112.5, *p* < 0.0001) (Table 4); that is, the group with a college degree exhibited the highest risk of progression, while illiterates had the lowest risk. Moreover, progression had no statistical correlations with the marriage state, family dementia history, personality tendency, family background, the lifecare mode, diabetes, smoking history, alcohol drinking history, and alcohol intake. Additionally, the positive end was related to more children (*F* (2, 1221) = 16.7, *p* < 0.001) (Figure 2(a)), lower height (*F* (2, 1221) = 4.52, *p* = 0.01), weight (*F* (2, 1221) = 6.61, *p*=0.001), and slightly to lower BMI levels (*F* (2, 1221) = 2.99, *p* = 0.05) (Figures 2(b)–2(d)). However, it is still early to tell some parameters can be risk factors. For example, we cannot distinguish the actual low height and the height loss due to osteoporosis and malnutrition. Next, the past occupation might decide the cognition impairment outcomes at the elderly stage (*χ*^2^ = 48.8, *p* < 0.0001) (Table 5); that is, public servants had an extremely high risk of cognition impairment progression. Another interesting finding is that the income level was positively related to progression (*χ*^2^ = 48.5, *p* < 0.0001) (Table 6).

**Figure 3:**
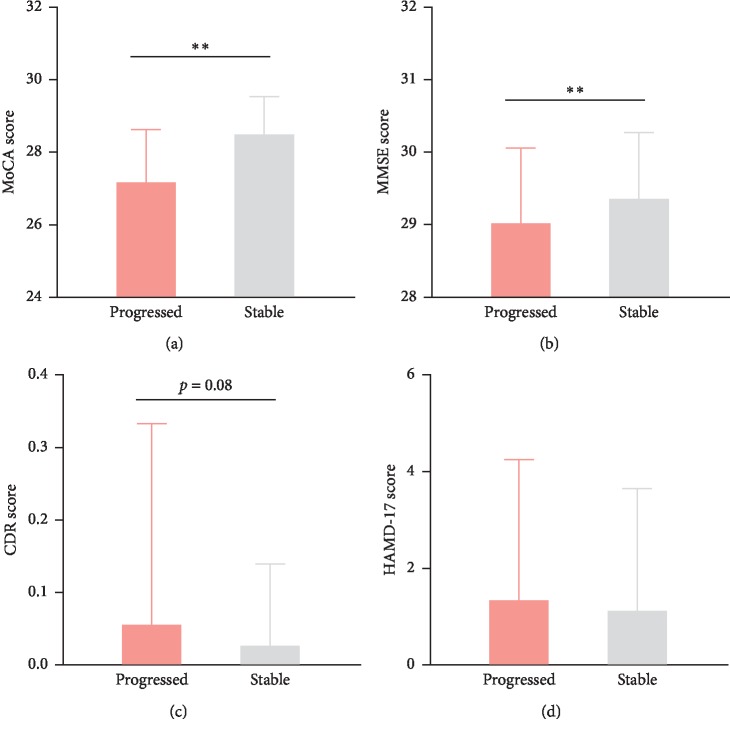
Different cognitive function test scores between outcome groups in 2017 among the healthy participants in 2011. (a) MoCA score. (b) MMSE score. (c) CDR score. (d) HAMD-17 score. ^*∗∗*^*p* < 0.01.

**Figure 4:**
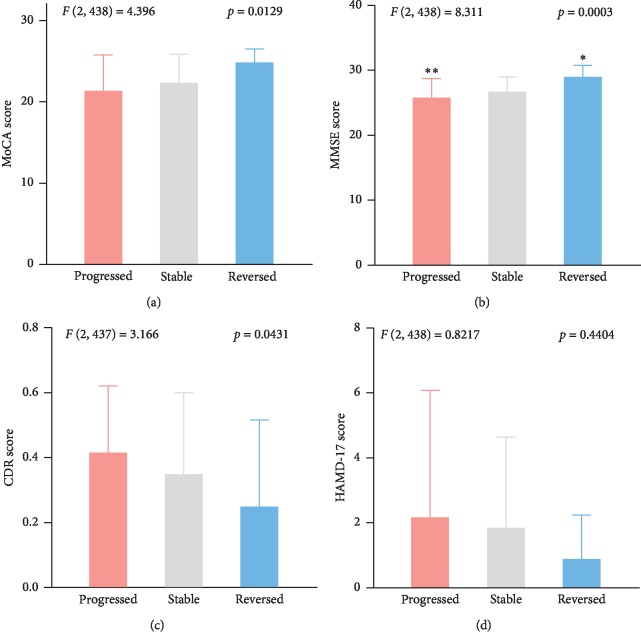
Different cognitive function test scores among three outcome groups in 2017 among the MCI participants in 2011. (a) MoCA score. (b) MMSE score. (c) CDR score. (d) HAMD-17 score. ^*∗*^*p* < 0.05 vs Stable, ^*∗∗*^*p* < 0.01 vs Stable.

In general, the progression outcomes were negatively related to the cognition impairment level in 2011 (not shown). However, this single regularity cannot help to predict the outcomes 6 years later. Therefore, we analyzed two groups of participants (healthy and MCI), respectively. As Figure 3 shows, for healthy cases, the progressed subgroup had poorer early MoCA (Figure 3(a)) and MMSE (Figure 3(b)) scores compared to the stable subgroup (*p* < 0.01). However, there were no differences in HAMD-17 and CDR scores (Figures 3(c) and 3(d)), as well as the ADL score (not shown). For the MCI group (Figure 3), MoCA (Figure 4(a)) and MMSE (Figure 3(b)) were also positively correlated with good outcomes, and the CDR score was positively related to progression outcomes (Figure 3(c)). Again, HAMD-17 showed no significant relationship with the progression outcomes (Figure 3(d)).

### 3.4. Factors in Prediction of MCI-Dementia and Healthy-Dementia Transformation

**Figure 5:**
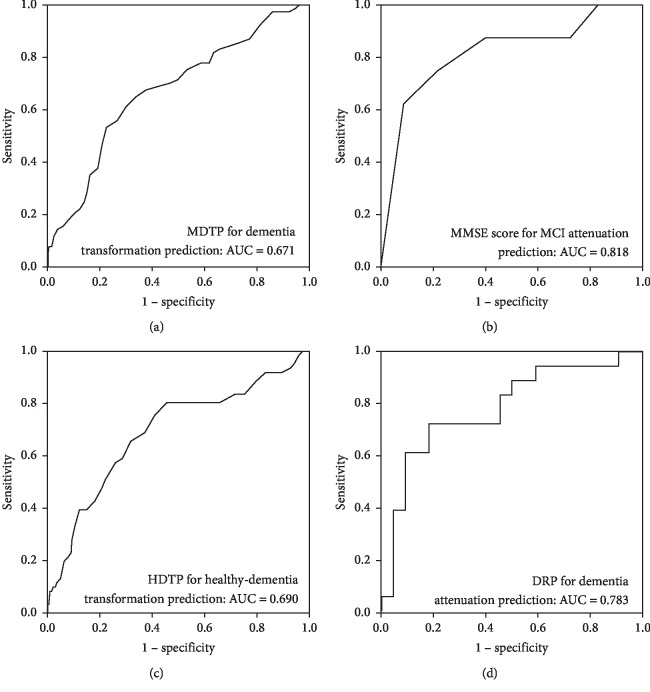
ROC curve for prediction of MCI-dementia and healthy-dementia transformation. (a) For MCI patients, a variable (MCI-dementia transformation predictor, MDTP) was calculated as: MDTP = Age − 2 ∗ MMSE score. (b) For MCI patients, using the variable MMSE score alone can specifically predict the attenuation outcome. (c) For healthy ones, a variable (healthy-dementia transformation predictor, HDTP) was calculated: HDTP = Age − 3 ∗ MMSE score − 2 ∗ MoCA score. (d) For those who were diagnosed dementia in 2011, the variable (dementia reversion predictor, DRP): DRP = MoCA score + 4.4 ∗ Children number − 10.1 ∗ Income group can help to indicate a positive end.

Finally, the above correlated factors were used together to construct models for dementia development towards both healthy and MCI cohorts. For MCI patients, a new variable (MCI-dementia transformation predictor, MDTP) was calculated as follows: MDTP = age − 2 ∗ MMSE score. This index provided a ROC curve with AUC around 0.671 (Figure 5(a)), with a sensitivity of 0.65 and a specificity of 0.66 at the cutoff threshold of 17.7. Moreover, using the variable MMSE score alone can specifically predict the attenuation outcome (AUC = 0.818, cutoff threshold = 29.5, sensitivity = 0.625, and specificity = 0.912) (Figure 5(b)). For healthy ones, we also constructed a variable (healthy-dementia transformation predictor, HDTP): HDTP = age − 3 ∗ MMSE score − 2 ∗ MoCA score, which exhibited an AUC of 0.690 in the ROC curve (with a sensitivity of 0.803 and a specificity of 0.544 at the cutoff value of −76.5) (Figure 5(c)). Additionally, using the variable (dementia reversion predictor, DRP) calculated as follows: DAP = MoCA score + 4.4 ∗ children number − 10.1 ∗ income group, the positive end might be indicated for those who were early diagnosed dementia (dementia group in 2011) (AUC = 0.783, cutoff threshold = 7.15, sensitivity = 0.722, and specificity = 0.818) (Figure 5(d)), which suggests that more children and better performance in the MoCA score may benefit for dementia recovery.

## 4. Discussion

In the present study, we enrolled 1229 elderly subjects to survey the features of MCI progression during more than six years. The main findings were as follows: the progression outcomes were different between sexes and among age brackets, education degrees, occupations types, and income levels; different progression groups had distinct children numbers, heights, and body weights; the positive ends six years later were positively related to better performance in the MoCA and MMSE scales. Moreover, we constructed some variables like MDTP (MCI-dementia transformation predictor), HDTP (healthy-dementia transformation predictor), and DRP (dementia reversion predictor) which might be helpful to predict outcomes.

So far, there have been some follow-up studies about cognitive changes of elderly MCI population, which have proposed the prognostic value of factors like nutritional status, transient ischemic attack, COPD status, diastolic dysfunction, and vascular risk factors [21–26]. Consistently, the gender differences and educational influences in MCI progression were reported by other teams [27–30]. Generally, female gender has a protective role because of the hormonal status. However, our work firstly exhibited some original indicators which might influence the development of MCI and dementia during a period of 6 years, such as occupations types, income levels, and children numbers. It is reasonable that the higher children number means more family interaction which counteracts cognition decline.

Among the above four scales, MoCA and MMSE were not only widely used but also recognized as efficient tests for cognition impairment probing; it has been widely reported that MoCA is superior to MMSE in discriminating between MCI and healthy individuals [31–33]. For dementia cases, MoCA and MMSE were similar, but MoCA distributes MCI cases across a broader score range with less ceiling effect [34]. We here used four scales to assess the cognition impairment, and as expected, MoCA and MMSE were efficient tools as they demonstrate some differences not observed in CDR and HAMD-17 (Figures 3 and 4). However, for the first time, we found they had different significances in healthy and MCI groups regarding the later progression. MoCA exhibited its efficacy in healthy individuals' progression (Figures 3(a) and 3(b)) and MMSE showed significance in the MCI cohort (Figure 4(a) and 4(b)). Moreover, the predictors (MDTP, HDTP, and DAP) can be easily calculated by just age and the scores of these two scales, which suggests that they have advantages in different aspects and can be applied for both distinguishing and predicting cognition impairment. Similar to our results, other independent studies also claimed their values as predictors of MCI progression, e.g., memory index score in MoCA was reported as an indicator of conversion from MCI to Alzheimer's disease [35]. As Figure 5(b) shows, using only MMSE too predict MCI attention reached an AUC of 0.818. This superiority has not been observed in other scales and never been reported, which highlight the clinical usefulness of MMSE. Nevertheless, the proportion of MCI improvement was small, and a higher efficacy in MDTP or HDTP is urgently needed.

Still, some limitations of our study deserve mention. First, we mainly focused on the total scores of the two scales and have not yet surveyed the diagnostic and prognostic values of different dimensions (specific subtests) in each scale. For example, word repetition, inverse digits, serial 7, phrases, verbal fluency, abstraction, and word recall in MoCA are known to be useful tools in distinguishing MCI and healthy individuals [36]. Additionally, we have not found enough consistent evidences to support the intriguing finding in our results that income levels were positively correlated with progression. It is still early to tell the underlying mechanism so far.

In conclusion, the MCI progression outcomes were associated with sex, age, education degrees, occupations types, income level, children number, height, and weight. MoCA and MMSE scales are efficient supporting tools to predict the progression outcomes, especially when combined with the demographic data.

## Figures and Tables

**Table 1:** Comparison of demographic characteristics and physical conditions between the lost cohort and the followed-up cohort.

Parameters	Followed-up (*n* = 1229) (%)	Lost (*n* = 1609) (%)	*t* or *χ*^2^	*p*
*Sex*			0.005	0.941
Male	424 (34.5)	589 (35.0)		
Female	805 (65.5)	1016 (65.0)		

*Years of education*			0.002	0.961
<12 years	907 (73.8)	1186 (74.0)		
≥12 years	322 (26.2)	415 (26.0)		

Age	67.0 ± 7.2	69.0 ± 8.2	7.106	0.001^*∗∗*^

*Age bracket*			27.723	0.001^*∗∗*^
<75	1001 (81.4)	1133 (70.4)		
≥75	228 (18.6)	476 (29.6)		

*In marriage*			8.823	0.003^*∗∗*^
No	205 (16.7)	341 (21.6)		
Yes	1024 (83.3)	1266 (78.4)		

*Past occupation style*			0.088	0.767
Brainwork	289 (23.5)	380 (23.6)		
Physical work	940 (76.5)	1229 (76.4)		

*Family background*			1.331	0.722
Live alone	110 (8.8)	158 (9.9)		
Nuclear family	675 (54.2)	844 (52.6)		
Stem family	456 (36.6)	597 (37.2)		
Other	5 (0.4)	5 (0.3)		

Children number	2.0 (1.0, 2.0)	2.0 (1.0, 2.0)	3.594	0.001^*∗∗*^

Height (m)	1.60 (1.56, 1.66)	1.60 (1.56, 1.67)	0.730	0.466

Weight (kg)	62.0 (55.0, 70.0)	62.0 (55.0, 70.0)	0.667	0.505

BMI (kg/m^2^)	23.88 (21.78, 25.95)	23.82 (21.55 25.97)	0.585	0.558

*Income (month)*			66.018	0.001^*∗∗*^
<minimum wage	238 (19.1)	236 (14.7)		
≤0.5^*∗*^ per capita wage	552 (44.2)	721 (45.0)		
≤per capita wage	441 (35.3)	619 (38.7)		
>per capita wage	17 (1.4)	25 (1.6)		

*Personality tendency*			0.864	0.649
Introvert	275 (22.1)	372 (23.2)		
Extrovert	443 (35.6)	546 (34.1)		
Middle type	527 (42.3)	683 (427)		

*Smoking history*			1.290	0.252
Yes	184 (15.0)	236 (14.7)		
No	1040 (85.0)	1369 (85.3)		

*Drinking history*			3.798	0.434
Yes	140 (11.6)	162 (10.2)		
No	1070 (88.4)	1424 (89.8)		

*Memory-loss complaint*			0.091	0.764
Yes	941 (75.5)	1202 (75.0)		
No	305 (24.5)	400 (25.0)		

*Family dementia history*			0.405	0.524
No	1121 (90.8)	1431 (90.1)		
Yes	114 (9.2)	158 (9.9)		

*Type-2 diabetes*			1.825	0.177
Yes	132 (10.7)	195 (12.3)		
No	1102 (89.3)	1385 (87.7)		

*Hypertension*			0.942	0.086
Yes	585 (47.1)	703 (43.9)		
No	656 (52.9)	898 (56.1)		

*Hyperlipidemia*			1.248	0.264
Yes	85 (6.9)	127 (8.0)		
No	1148 (93.1)	1458 (92.0)		

**Table 2:** Differences in progression outcomes between sexes.

Sex	Progressed	Stable	Alleviated	*χ* ^2^	*p*
Male	285	140	6	14.1	0.001
%	66.1	32.5	1.4		
Female	447	323	28		
%	56.0	40.5	3.5		

**Table 3:** Differences in progression outcomes among age bracket.

Age	Progressed	Stable	Alleviated	*χ* ^2^	*p*
50–59	126	75	3	29.247	<0.001
%	61.8	36.8	1.5		
60–69	368	219	10		
%	61.6	36.7	1.7		
70–79	206	147	17		
%	55.7	39.7	4.6		
80–89	32	21	3		
%	57.1	37.5	5.4		
90–99	0	1	1		
%	0.0	50.0	50.0		

**Table 4:** Differences in progression outcomes among education degrees.

Education degree	Progressed	Stable	Alleviated	*χ* ^2^	*p*
Illiterate	31	98	10	112.5	<0.001
%	22.3	70.5	7.2		
Primary school	173	116	5		
%	58.8	39.5	1.7		
Middle school	298	161	15		
%	62.9	34.0	3.2		
College	173	53	3		
%	75.5	23.1	1.3		
Bachelor	33	18	1		
%	63.5	34.6	1.9		
Postgraduate	24	17	0		
%	58.5	41.5	0.0		

**Table 5:** Differences in progression outcomes among past occupations.

Occupation	Progressed	Stable	Alleviated	*χ* ^2^	*p*
Technician	111	50	5	48.8	<0.001
%	66.9	30.1	3.0		
National cadre	37	16	0		
%	69.8	30.2	0.0		
Public servant	32	11	0		
%	74.4	25.6	0.0		
Commerce	13	9	0		
%	59.1	40.9	0.0		
Service staff	10	5	0		
%	66.7	33.3	0.0		
Peasant	50	83	8		
%	35.5	58.9	5.7		
Worker	363	219	15		
%	60.8	36.7	2.5		
Other	115	66	6		
%	61.5	35.3	3.2		

**Table 6:** Differences in progression outcomes among income levels.

Monthly income	Progressed	Stable	Alleviated	*χ* ^2^	*p*
<minimum wage	94	125	12	48.5	<0.001
%	40.7	54.1	5.2		
≤0.5^*∗*^ per capita wage	348	183	14		
%	63.9	33.6	2.6		
≤per capita wage	279	149	7		
%	64.1	34.3	1.6		
>per capita wage	11	5	1		
%	64.7	29.4	5.9		

## Data Availability

The data used to support the findings of this study are available from the corresponding author upon request.
